# Novel and emerging sources of *Clostridioides difficile* infection

**DOI:** 10.1371/journal.ppat.1008125

**Published:** 2019-12-19

**Authors:** Nicholas A. Turner, Becky A. Smith, Sarah S. Lewis

**Affiliations:** 1 Duke University Medical Center, Department of Medicine, Division of Infectious Diseases, Durham, North Carolina, United States of America; 2 Duke University Medical Center, Duke Center for Antimicrobial Stewardship and Infection Prevention, Durham, North Carolina, United States of America; Tufts Univ School of Medicine, UNITED STATES

## Introduction

*Clostridioides difficile* causes more healthcare-associated infections in the United States than any other pathogen, with an estimated 500,000 infections and 29,000 deaths per year [1]. Although *C*. *difficile* first emerged as a healthcare-associated pathogen, infections are increasingly acquired in the community [2]. Genomic epidemiology studies have shown that clinical *C*. *difficile* isolates are more diverse than previously presumed and point toward unidentified environmental reservoirs [3]. An improved understanding of *C*. *difficile’s* epidemiology is essential for future infection prevention efforts, both within healthcare settings and in the community. Here, we provide an overview of the changing epidemiology of *C*. *difficile* and current evidence for novel sources of transmission.

## How is *C*. *difficile* epidemiology changing?

*C*. *difficile* first gained attention in the early 2000s, with a rapid increase in healthcare-associated *C*. *difficile* infection (CDI) driven by the emergence of the hypervirulent North American pulsed-field gel electrophoresis (PFGE) type 1 (NAP1)/027 (PCR ribotype 027) strain [4]. High-level fluoroquinolone resistance likely contributed to the rise of NAP1 incidence in the early 2000s, whereas production of toxin with a higher binding affinity and more rapid cellular entry likely drove the excess mortality rates seen with NAP1 [5,6]. Although total CDI rates have not significantly fallen over the past decade, the prevalence of NAP1 has declined in some regions [4,7]. Over the same time period, an increasing number of CDI cases have been observed among individuals without significant prior hospital contact (referred to as community-acquired CDI, or CA-CDI) [2,8].

For a variety of reasons, the study of CA-CDI is more difficult than healthcare-associated CDI. First, there are few population-based studies of CDI incidence, and the majority of data comes from hospital-based surveillance of CDI among patients admitted to hospitals [1,2,9]. Hospital-based surveillance programs typically follow the National Healthcare Safety Network definitions, which categorize cases of CDI diagnosed within the first 3 days of hospitalization as community-acquired unless the patient was admitted to the same hospital within the preceding 4 weeks. Failure to account for contact with other types of healthcare facilities may misclassify healthcare-associated cases as community-acquired. Robust population-level studies of CDI risk are hindered by difficulties in defining the true population at risk, as well as accurately tracking all potentially relevant outpatient healthcare contacts. One of the major issues with defining populations at risk for CA-CDI is a lack of centralized testing or surveillance. Because patients are able to present to urgent cares, primary care offices, emergency rooms, and hospitals, often all belonging to different healthcare networks, it is extremely difficult to determine how many cases are occurring within a particular community.

## What risk factors are associated with CA-CDI?

Similar to healthcare-associated *C*. *difficile*, case-control studies consistently associate antibiotic receipt with increased risk of CA-CDI [10–12]. Proton pump inhibitors (PPI) have a more controversial role in CDI risk. Although several epidemiologic studies have linked PPI exposure with healthcare-associated CDI, PPI receipt has not been consistently associated with increased risk among CA-CDI [13, 14].

Contact with outpatient healthcare facilities (e.g., clinics, urgent cares, dentists) is also common among patients with CA-CDI [2]. One recent multicenter case-control study conducted by the Centers for Disease Control (CDC) found that >80% of CA-CDI cases had contact with an outpatient healthcare facility in the preceding 12 weeks [10]. It remains unclear whether the healthcare facilities themselves play a direct role in *C*. *difficile* transmission or if this simply reflects confounding in which chronically ill patients are both at higher risk of *C*. *difficile* and more likely to require frequent care.

Regardless of the exact sources involved, reservoirs for CDI outside of the hospital pose substantial challenges to prevention efforts given that most current initiatives to prevent CDI focus on interventions made within hospitals.

## How has molecular epidemiology improved our understanding of *C*. *difficile* transmission?

*C*. *difficile* persists in the environment, is resistant to many routine disinfectants, and causes outbreaks among epidemiologically linked patients. For these reasons, it has been presumed that *C*. *difficile* is primarily spread from patient to patient through contaminated hands of healthcare workers, shared equipment, and environmental surfaces. However, current genomic data point toward far more diverse sources of acquisition. A landmark United Kingdom study using whole-genome sequencing found that 45% of *C*. *difficile* isolates from hospitalized patients were genetically distinct from all other cases—consistent with a large and extremely diverse reservoir for *C*. *difficile* acquisition [3].

Although many novel sources for *C*. *difficile* transmission have been proposed, few have been rigorously confirmed. The sheer diversity of circulating *C*. *difficile* strains challenges efforts to untangle patterns of CDI spread. Aside from revealing much greater diversity than previously anticipated, molecular epidemiologic methods inform many of the potential novel sources for *C*. *difficile* transmission, as follows.

## What are the potential novel sources of *C*. *difficile*?

### Spread between hospitals and the community

Several large-scale molecular epidemiologic studies have confirmed overlap between *C*. *difficile* strains circulating within the community and among healthcare-acquired cases [9,15]. One large UK study conducted ribotyping on >700 *C*. *difficile* isolates from a multicenter network and found relatively balanced occurrence of most ribotypes between community and healthcare settings [9]. A similar study in Australia arrived at the same conclusion, with nearly 80% of ribotypes occurring among both community- and healthcare-acquired cases [15]. More recently, whole-genome sequencing has confirmed the occurrence of genetically related *C*. *difficile* isolates among both community- and healthcare-acquired cases [16]. The same study evaluated relevant healthcare contact in the 12 months preceding CDI and found that nearly all presumed CA-CDI cases had some hospital contact in the preceding year. These data suggest that even distant past healthcare contact may be relevant to subsequent CDI [16]. Despite clear evidence that *C*. *difficile* transmission can occur between the healthcare and community environments, existing studies are unable to determine the directionality of transmission. Similarly, even though healthcare contact is frequently associated with CA-CDI, it remains unclear if this reflects patients who are actually at elevated risk because of multiple chronic health problems or if contact with healthcare is truly what is driving the risk.

### Role of asymptomatically colonized hosts

*C*. *difficile* carriage rates range from 5.7% to 11.1% among screened inpatients, 7.6% to 24.0% among long-term acute care facility (LTAC) residents, and 16% to 40% among infants (notably, although infants carry *C*. *difficile* at high rates, they do not develop the disease) [17]. Because asymptomatic carriers can shed *C*. *difficile* spores in their stool, environmental contamination rates can actually be similar to those with active CDI. Although mathematical modeling studies suggest that implementing contact isolation (placing patients in a single room and requiring healthcare workers to wear gowns and gloves during the patient encounter) for carriers may help to reduce *C*. *difficile* transmission rates, clinical data supporting the effectiveness and cost-effectiveness of contact isolation for *C*. *difficile* carriers are lacking [18]. Molecular epidemiologic studies attribute a relatively small minority of transmission events to carriers [3].

### Nonhospital healthcare contacts

Several large-scale cohort and geospatial studies consistently link nursing home or long-term care facility exposure to increased CDI risk [19]. Given high CDI incidence rates and a high likelihood of readmission to the hospital after developing CDI, expansion of infection prevention efforts to nursing and long-term care facilities is likely necessary for reducing the spread of CDI within healthcare networks.

### Household transmission

Microbiologic surveys of households with a resident family member recently having had CDI confirm widespread contamination. One large-scale case-contact study found an increased risk for CDI among household contacts of patients with CDI for up to 3 months after occurrence of the index infection [20]. Despite a clearly increased relative risk (RR for spouses of cases: 5.77–9.78), the absolute risk increase for CDI remained small, however (4.71–5.99 per 1,000). Other home surveys found high rates of *C*. *difficile* contamination across a range of household surfaces even without any known household CDI contacts [21].

### Environmental reservoirs

*C*. *difficile* has also been recovered from parks, lawns, soil, beaches, river water, pools, and municipal wastewater, though the relevance of these reservoirs to transmission remains unclear [22,23]. One UK study conducted whole-genome sequencing on regional pairs of clinical and wastewater *C*. *difficile* isolates, finding both a high degree of genetic diversity overall but also significant overlap between clinical and environmental isolates [22]. Whether environmental contamination is a true source for disease or is simply a consequence of shedding by carriers or infected individuals remains unknown.

### Zoonotic potential

*C*. *difficile* carriage has been well documented for a wide range of animals, including pets, swine, and cattle [24,25]. Ribotype 078 predominates among livestock, and the same ribotype has been associated with community-acquired human cases in some regions. High rates of CDI carriage have also been reported among livestock workers, particularly swine handlers [26]. One Dutch study conducted whole-genome sequencing on *C*. *difficile* isolates from pigs, human CDI cases, and asymptomatically colonized pig farmers. Just under half of paired *C*. *difficile* isolates from pig farmers and their respective animals were identical, confirming transmission between animals and humans [27]. Within the same study, several clinical isolates were found to be identical to pig isolates, implicating human‒animal transmission in true disease, not just colonization. Subsequent phylogenetic surveys applying whole-genome sequencing to ribotypes 078 and 014 (two isolates well described among both human and animal hosts) found a high degree of co-clustering between human and animal isolates, consistent with frequent bidirectional transmission [28].

Which factors are driving the emergence and transmission of particular zoonotic strains has become a particularly active area of interest, currently focused on feed supplements and agricultural antibiotics. Use of trehalose as a feed supplement in swine has been suggested as a risk factor for *C*. *difficile* carriage, based in part on genomic and metabolic data. A point mutation in the trehalose repressor of ribotype 027 increases sensitivity to trehalose concentration, whereas ribotype 078 has acquired a cluster of genes that enhance metabolism of trehalose at low concentrations [29]. A recent UK study also found evidence for increasing prevalence of tetracycline resistance among ribotype 078 isolates, suggesting a potential role for the use of agricultural antibiotics in selection of emerging *C*. *difficile* strains [30].

### Foodborne

With high rates of carriage among livestock, it is not surprising that contamination of meat products with *C*. *difficile* has been reported, including pork, beef, poultry, and processed meats [31,32]. However, no foodborne outbreaks have been described, and molecular surveys show little to no overlap between meat-associated and human disease strains.

## Conclusions

Overall, the high degree of diversity among *C*. *difficile* isolates, increasing evidence of transmission outside of the hospital environment, and multiple potential sources of infection challenge current infection control efforts. Improving our understanding of community-acquired *C*. *difficile* epidemiology will require detailed prospective collection of wide-ranging exposure-related data on a large scale, coupled with whole-genome sequencing. With the additional issues of widespread outpatient healthcare contact, asymptomatic carriage, and long-term environmental persistence of spores, even the basic distinction between community- versus healthcare-associated CDI may become less relevant with time. Given the challenges posed by current evidence of interspecies transmission and environmental reservoirs of *C*. *difficile*, future research in C. difficile prevention will require an integrative multidisciplinary approach, as exemplified by the OneHealth concept.
